# Supplementary material for: Indoloquinoline-Mediated Targeted Downregulation of KRAS through Selective Stabilization of the Mid-Promoter G-Quadruplex Structure
Source: Genes (Basel). 2022 Aug 13;13(8):1440. doi: 10.3390/genes13081440 (PMC9408018; doi:10.3390/genes13081440)
Supplement: Supplementary file 1 [file genes-13-01440-s001.zip › genes-1778216-supplementary.pdf]

Supplemental Figures:

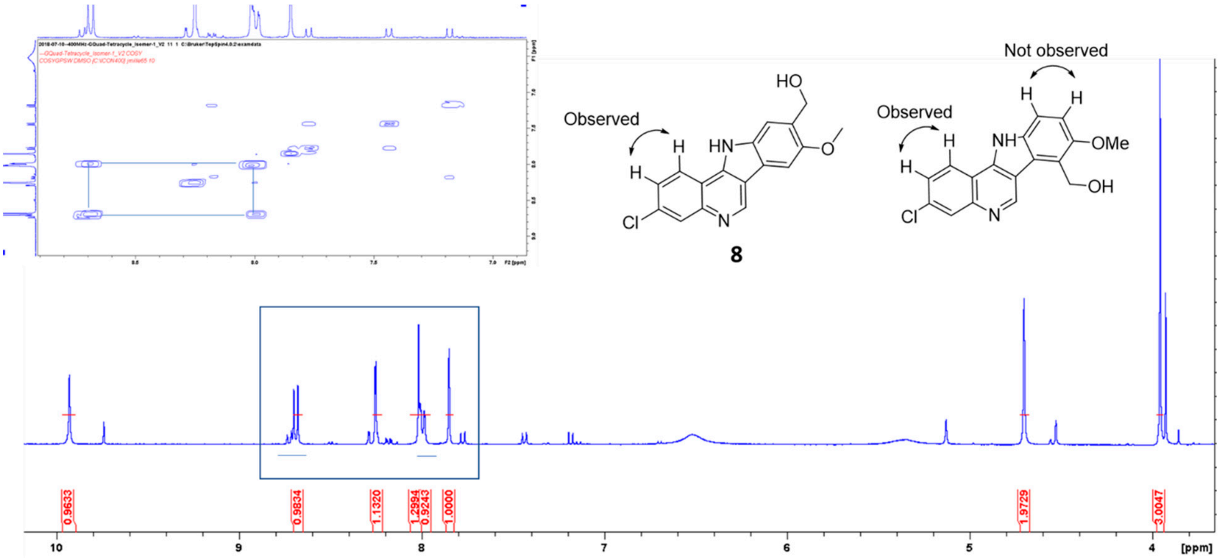

Figure S1. Verification of the structure of compound **8**.

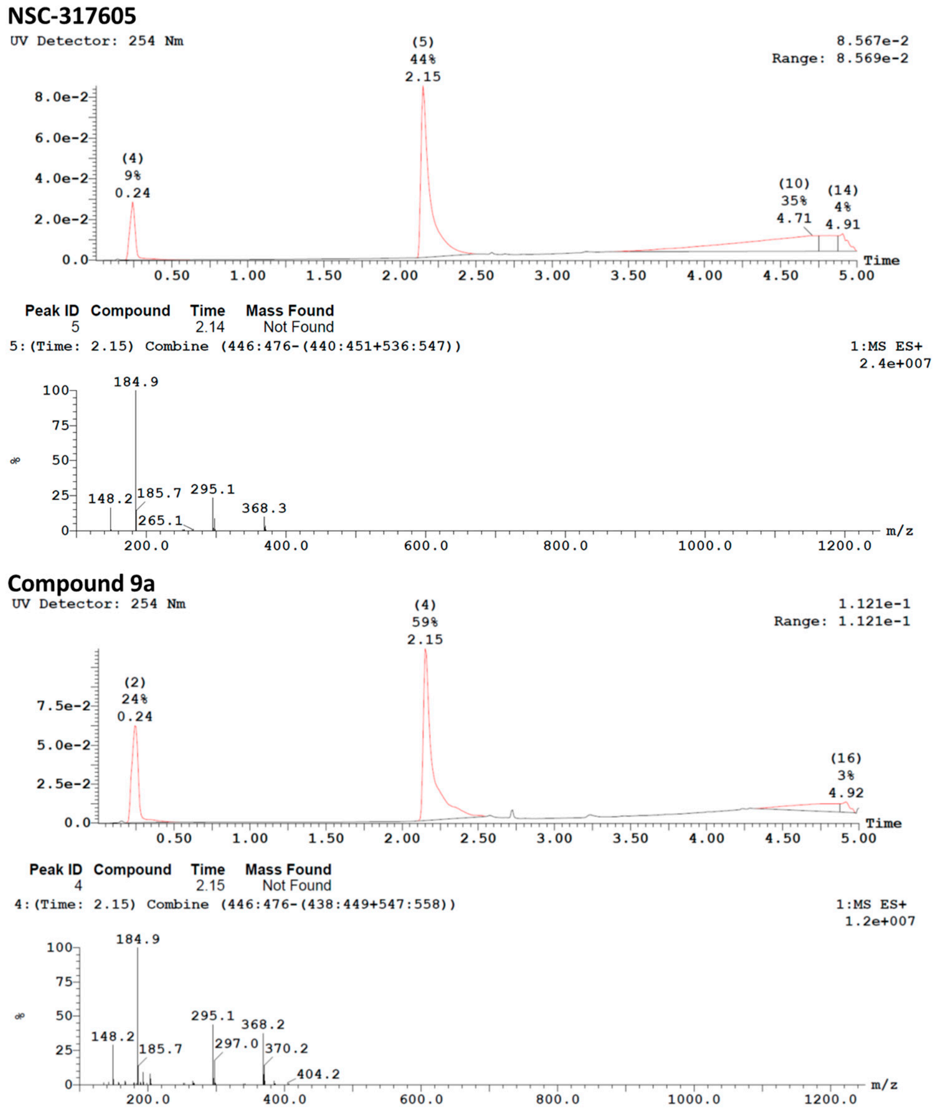

Figure S2. Confirmation of identify between compound **9a** to NSC 317605. (note the peak at 0.24 is the solvent front DMF used for injection)

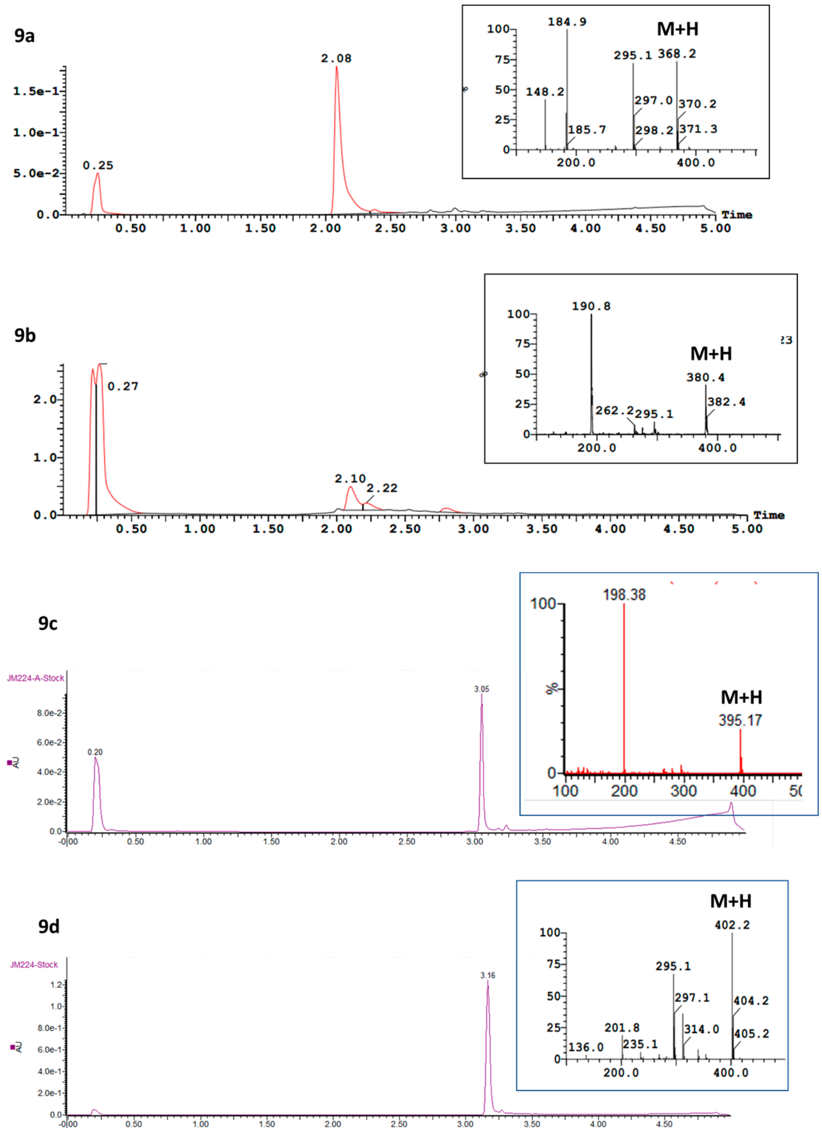

Figure S3. LCMS confirmation of purity and identity for compounds for **9a-9d.**

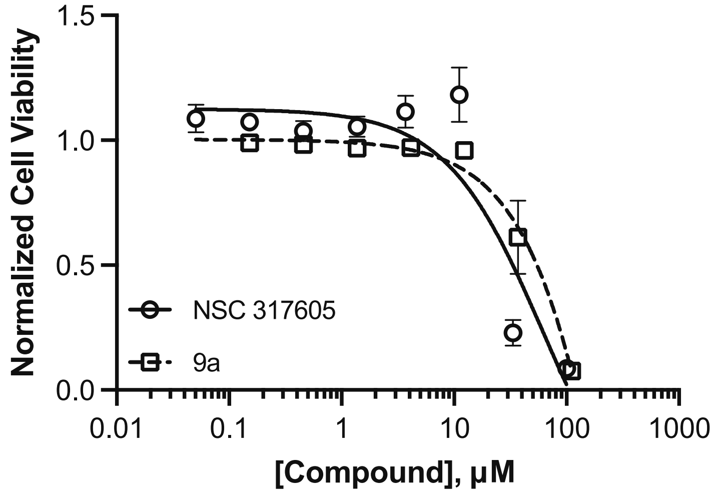

Figure S4. Comparison of cytotoxicity of **9a** to NSC 317605 in AsPc-1 cells at 72 hr. A comparison of IC50′s by Extra sum-of-squares F Test highlight no significant difference between the IC_{50}s.
